# FBXW7 suppresses gastric cancer development and progression via ubiquitination-mediated degradation of MMP2

**DOI:** 10.3389/fimmu.2026.1877833

**Published:** 2026-08-04

**Authors:** Changjiang Hao, Shengping Jiang, Songpu Wu, Weiwei Li, Jilv Peng, Bo Qin, Jinlan Li, Yong Tan, Kun Qian, Hongsheng Jiang, Yi Li, Guoquan Huang

**Affiliations:** 1 Hubei Key Laboratory for Translational Research in Traditional Chinese Medicine, Hubei Selenium and Human Health Institute, Department of Colorectal and Anal Surgery, The Central Hospital of Enshi Tujia and Miao Autonomous Prefecture, Hubei Minzu University, Enshi, China; 2 Hubei Provincial Key Lab of Selenium Resources and Bioapplications, Enshi, China; 3 Modern Research Center for Traditional Chinese Medicine, Key Laboratory of Research and Utilization of Bioactive Components in Famous shanxi Medicinal Materials, shanxi University, Taiyuan, China; 4 Department of Emergency Surgery, Union Hospital, Tongji Medical College, Huazhong University of Science and Technology, Wuhan, China; 5 Division of Nephrology, The Central Hospital of Enshi Tujia and Miao Autonomous Prefecture, No.158 Wuyang Avenue, Enshi City, Hubei, China; 6 State Key Laboratory of Biocatalysis and Enzyme Engineering, Hubei Key Laboratory of Industrial Biotechnology, School of Life Sciences, Hubei University, #368 Youyi Road, Wuhan, China; 7 Department of Gastrointestinal Surgery, The First Affiliated Hospital of Chongqing Medical University, Chongqing, China; 8 Department of Hepatobiliary Surgery, The Central Hospital of Enshi Tujia and Miao Autonomous Prefecture, Enshi, China

**Keywords:** FBXW7, gastric cancer, MMP2, single-cell sequencing, ubiquitination

## Abstract

**Background:**

Gastric cancer (GC) remains a major cause of cancer-related mortality, highlighting the need to clarify the stromal and post-translational mechanisms underlying its progression.

**Methods:**

We integrated single-cell RNA sequencing, independent transcriptomic cohorts, clinical tissue analyses, functional assays, co-immunoprecipitation (Co-IP), multiple immunofluorescence labeling, and a xenograft model to investigate the cellular distribution, biological function, and mechanisms of regulation for matrix metalloproteinase 2 (MMP2) in GC.

**Results:**

Single-cell analysis of paired tumor and adjacent normal tissues suggested an increased relative abundance of fibroblast populations and higher extracellular matrix-associated activity in GC tissues. MMP2 was preferentially expressed in cancer-associated fibroblasts, particularly in transcriptionally defined universal fibroblast-like and myofibroblastic CAF states. Trajectory analysis further suggested that the MMP2-high universal fibroblast-like population occupies an early and potentially transitional position along the fibroblast-state continuum. These findings were supported by an independent single-cell dataset, TCGA-STAD analysis, and tissue staining. In CAF–gastric cancer cell co-culture assays, CAFs enhanced tumor-cell migration and invasion, while modulation of the FBXW7–MMP2 axis altered these phenotypes. MMP2 overexpression promoted GC cell proliferation, migration, and invasion, whereas MMP2 silencing had the opposite effects. Mechanistically, FBXW7 interacted with the intracellular pool of MMP2, enhanced its K48-linked polyubiquitination, and reduced MMP2 protein stability through a proteasome-dependent process. FBXW7 overexpression attenuated MMP2-associated malignant phenotypes *in vitro* and restrained MMP2-driven tumor growth *in vivo*. Clinical tissue analyses further showed an inverse association between FBXW7 and MMP2 expression, while elevated MMP2 was associated with unfavorable survival.

**Conclusion:**

Our findings identify MMP2-high fibroblast states as an important stromal feature of GC and reveal FBXW7-associated regulation of intracellular MMP2 stability as a potential therapeutic vulnerability.

## 1 Introduction

Gastric cancer (GC) ranks as the fifth most common malignancy and the fourth leading cause of cancer-related death worldwide, posing a major global health burden (1, 2). In China, GC ranks third in terms of incidence and second in mortality, with a five-year survival rate of merely 35.1% (3–5). Alarmingly, approximately half of the global deaths attributed to GC occur in China, highlighting the urgent necessity for innovative therapeutic strategies. Despite advancements in surgical and systemic treatments, the molecular mechanisms driving GC progression remain inadequately characterized, impeding the development of targeted interventions.

The tumor microenvironment (TME) plays a critical role in gastric carcinogenesis (6), with CAFs acting as key regulators of tumor growth, invasion, and metastasis (7, 8). Matrix metalloproteinases (MMPs), particularly MMP2, facilitate ECM remodeling, thereby promoting tumor dissemination (9, 10). Although elevated MMP2 levels are associated with poor prognosis in various cancers, the regulatory mechanisms governing MMP2 in GC, particularly its post-translational modifications, remain poorly understood. While ubiquitination is a crucial regulator of protein turnover, the specific E3 ubiquitin ligase targeting MMP2 in GC has yet to be identified.

F-box and WD repeat domain-containing 7 (FBXW7) is a well-established tumor suppressor that serves as the substrate-recognition component of the SCF (SKP1-CUL1-F-box) ubiquitin ligase complex (11–13). It promotes the polyubiquitination and subsequent proteasomal degradation of oncogenic proteins, including c-Myc, cyclin E, and NOTCH, thereby suppressing tumorigenesis (14). Our previous research has demonstrated that FBXW7 plays a pivotal role in suppressing GC by preventing primary tumor development and peritoneal metastasis (15). Notably, FBXW7 deficiency, which is common in GC, is associated with chemotherapy resistance and more aggressive cancer phenotypes (16, 17). Previous studies have shown that E3 ubiquitin ligases and deubiquitinases (dub) play a crucial role in the development of colorectal cancer. The mechanism involves influencing the Wnt/β-catenin and NF-κB signaling pathways to exert regulatory effects (18). However, the potential regulatory role of FBXW7 on MMP2 and the clinical implications of this interaction remain unexplored.

In this study, we utilize scRNA-seq and The Cancer Genome Atlas (TCGA) cohort analyses to identify MMP2 as a CAF-specific effector, enriched in UFs and indicative of poor clinical outcomes. Functional assays reveal that MMP2 enhances GC cell proliferation, migration, and invasion, whereas its knockdown mitigates these oncogenic processes. Mechanistically, our findings reveal a previously unrecognized role of FBXW7 in mediating MMP2 polyubiquitination and proteasome-dependent degradation. The overexpression of FBXW7 leads to the destabilization of MMP2 without affecting its transcription, while the loss of FBXW7 results in the accumulation of MMP2 and exacerbates tumorigenesis. *In vivo* models confirm the FBXW7-MMP2 axis as a significant vulnerability in the progression of GC.

Our findings identify FBXW7 as a molecular inhibitor of MMP2-mediated malignancy, thereby highlighting the FBXW7-MMP2 axis as a viable therapeutic target. This study reveals a previously unrecognized post-translational regulatory mechanism for MMP2 and suggests FBXW7 expression as a potential prognostic biomarker for GC. Targeting this pathway could facilitate precision therapy for GC patients exhibiting dysregulated MMP2 activity.

## 2 Experimental model and study participant details

### 2.1 Human gastric cancer samples

For single-cell analysis, paired primary gastric cancer and adjacent normal tissues were obtainedfrom three patients diagnosed with gastric cancer at the Department of Gastrointestinal Surgery, the First Affiliated Hospital of Chongqing Medical University. Detailed clinical and pathological characteristics (including tumor stage, histological type, and patient demographics) are documented in **Supplementary Table 1**. The tissue chips (HColA180Su01) of 89 cases of human gastric cancer and adjacent normal tissues were purchased from shanghai Outdo Biotech Co. Ltd (shanghai, China), The patient ethics was approved by the Ethics committee of shanghai Xinchao Biotechnology Co., LTD. (ID: YB M-05-01, YB M-05-02). All patients were diagnosed as gastric cancer. In this study, tissue chip specimens were obtained from July 2010 to December 2010, followed up from June 2016 for 5.5 to 6 years. All samples were examined and approved by the Ethics Committee of shanghai Outdo Biotech Co. Ltd. The ethics number is: shYJS-CP-1910016. Ethical approval for this study was obtained from the Ethics Committee of the First Affiliated Hospital of Chongqing Medical University (ID: K2023-100), and all participants provided written informed consent prior to sample collection. None of the patients received neoadjuvant therapy before surgery.

### 2.2 Cell culture and reagents

Human gastric cancer cell lines (HGC-27, SNU-638, NUGC-3, MKN-45) and the normal gastric mucosal cell line GES-1 were obtained from the Cell Bank (procell) of Chinese Academy of Sciences and maintained in RPMI 1640 (C11885500BT, USA) or DMEM (C11995500BT, USA) medium supplemented with 10% fetal bovine serum (FBS) at 37 °C in a 5% CO_2_ incubator. The following antibodies were used for protein detection: anti-MMP2 (1:500, ab97779/ab92536, Abcam), anti-FBXW7 (1 µg/ml, ab109617, abcam), and anti-HA-Ub (1:500, 3724S, CST). Proteasome inhibitor MG132 (S2619, Selleck Chemicals, experimental concentration:20µM) and protein synthesis inhibitor cycloheximide (CHX; C7698, Sigma-Aldrich, experimental concentration:100µg/mL) were used for cellular treatments.

Construction of CAFs-gastric cancer cell co-culture system: 1. The lower layer cells (gastric cancer adherent cells): 2×10^5^ gastric cancer cells were seeded in each well of HGC-27 6-well plate, and the cells adhered stably after incubation overnight at 37°C.

Treatment of cells in the upper chamber: the upper chamber was paved with 1×10^5^ CAF and 1 mL medium. 3. Assemble the co-culture system: remove the 6-well plate of adherent gastric cancer cells, discard the old medium, and rinse once with PBS. The 0.4μm Transwell chamber with cells was smoothly placed into the well, and the lower layer was supplemented with fresh complete medium to the total liquid 2 mL (lower layer) and 1 mL (upper chamber). The liquid level did not exceed the chamber to separate the diaphragm, and the chamber was cultured in 37°C and 5% CO_2_ constant temperature incubator for 48 hours. 4. Sample collection treatment: the Transwell chamber was removed, the medium was directly aspirated from the lower hole, and the gastric cancer cells were washed with PBS and collected by trypsin digestion. Sheeting was used for wound healing and transwell assays.

### 2.3 Animal experiments

Subcutaneous tumor xenograft model Male BALB/c nude mice (4–6 weeks old) were purchased from Wuhan Myhalic Biotechnology Co., Ltd. All animal experiments were approved by the Ethics Review Committee of Enshi Tujia and Miao Autonomous Prefecture Central Hospital (Approval No. W202410002). All procedures were performed in accordance with relevant institutional and national guidelines and regulations for the care and use of laboratory animals. This study was conducted and reported in compliance with the ARRIVE guidelines (https://arriveguidelines.org).

To establish subcutaneous xenograft tumors, 1 × 10^6^ HGC-27 cells suspended in 100 μL of phosphate-buffered saline (PBS) were subcutaneously injected into the right dorsal flank of each mouse. Experiments were initiated when the tumor volume reached approximately 70 mm³. Mice were randomly assigned to three experimental groups (n = 5 per group) using a computer-generated randomization protocol: Group 1 (NC): OE-NC (negative control), Group 2 (MMP2-OE): OE-MMP2 (GenePharma, shanghai, China), Group 3 (Rescue): OE-MMP2 + OE-FBXW7. Briefly, cholesterol-modified OE-NC, OE-MMP2, or OE-MMP2+OE-FBXW7 were mixed with an *in vivo* transfection reagent (Engreen, Beijing, China) and dissolved in sterile saline to a total volume of 25 µL per mouse. Each nude mouse received multiple intratumoral injections at 1-day intervals. Tumor volume was measured with calipers and recorded to generate growth curves. Animals were humanely euthanized if tumor volume exceeded 1500 mm³ or body weight loss ≥20% prior to the endpoint. Mice were anesthetized using isoflurane inhalation with an induction concentration of 3-5% and a maintenance concentration of 1-2%. Euthanasia was performed via carbon dioxide inhalation at a flow rate of 10-30% of the cage volume per minute, which was maintained until respiratory arrest, followed by an additional 5 minutes to ensure death. After approximately 3 weeks of treatment, mice were euthanized using 3% isoflurane in oxygen, and tumor tissues were harvested for photography, weighing, immunohistochemistry, and western blot analysis.

### 2.4 Single-cell RNA-seq library construction and sequencing

Tissue samples were dissociated into single-cell suspensions using a mixture of 1 mg/mL collagenase IV (Gibco) and 0.1 mg/mL DNase I (Sigma) at 37 °C for 30 min, followed by filtration through a 70-μm cell strainer. Single-cell libraries were generated using the Singleron Biotechnologies GEXSCOPE^®^ platform (Nanjing, China) according to the manufacturer’s protocol. Briefly, approximately 10,000 cells were loaded onto the microfluidic chip for encapsulation, reverse transcription, and cDNA amplification. Libraries were constructed with unique barcode indices, and sequencing was performed on an Illumina NovaSeq 6000 platform (Illumina, USA) to generate 150-bp paired-end reads, with an average of 200 million reads per sample.

### 2.5 Data preprocessing and quality control for scRNA-Seq

Raw sequencing data were aligned to the human reference genome (GRCh38) using Cell Ranger (version 6.1.2). Downstream quality control and analyses were conducted in Python using Scanpy (version 1.9.6). Cells were retained if they satisfied all of the following criteria: 200-6,000 unique genes detected, total UMI count >500, and mitochondrial gene fraction <20%. Doublets were identified and removed using Scrublet. The retained cells were normalized to 10,000 counts per cell and log1p-transformed. The top 2,000 highly variable genes (HVGs) were selected using the ‘seurat_v3’ dispersion method for downstream analyses.

### 2.6 Cell clustering, annotation, and trajectory analysis

Clustering and dimensionality reduction: Principal component analysis (PCA) was performed using highly variable genes (HVGs), and the top 30 principal components were corrected for inter-sample batch effects using Harmony (version 0.1.9). The batch-corrected embedding was used to construct a k-nearest neighbor (kNN) graph, followed by Leiden community detection for unsupervised clustering. UMAP was applied for two-dimensional visualization.

Cell type annotation: Cell types were annotated based on canonical marker gene expression for each cluster, corroborated with SingleR automated annotation. Twelve major cell types were identified: B cells (CD79A, MS4A1), T cells (CD3D, CD8A), Macrophages (CD68, MRC1), Endothelial cells (PECAM1, VWF), Fibroblasts (COL1A1, DCN), myCAFs (ACTA2, POSTN), Pit mucous cells (MUC5AC, TFF1), Gland mucous cells (MUC6, TFF2), Parietal cells (ATP4A, ATP4B), Enteroendocrine cells (CHGA, NEUROD1), Enterocytes (FABP1, ALPI), and Stem cells (LGR5, OLFM4). An ECM/invasion activity module score was computed per cell as the mean scaled expression of curated ECM and MMP genes (COL1A1, COL3A1, FN1, MMP2, MMP14, TIMP1, ITGA5, ITGB1, LOXL2).

Trajectory inference: For fibroblast subtype analysis, fibroblast and myofibroblastic (myCAF) cells were re-extracted and re-clustered at higher resolution, yielding three subtypes: Universal Fibroblasts (UF), myCAFs, and inflammatory CAFs (iCAF). Developmental trajectories were reconstructed using partition-based graph abstraction (PAGA) implemented in Scanpy, which computes connectivity scores between cell partitions. Diffusion pseudotime (DPT) was then computed with UF cells designated as the root population based on their highest PAGA connectivity. Pseudotime density distributions were compared across subtypes to infer differentiation order.

### 2.7 Differential expression and functional enrichment analysis

Differentially expressed genes (DEGs) between cell types and fibroblast subtypes were identified using the Wilcoxon rank-sum test (Scanpy sc.tl.rank_genes_groups), with significance defined as adjusted *p*-value <0.05 and |log2 fold change| >0.5. Gene co-expression analysis used Spearman correlation coefficients computed pairwise across all fibroblast cells. GO Biological Process and KEGG pathway enrichment analyses for UF-specific upregulated genes were performed with gseapy (version 1.0.4) using the Enrichr API. Volcano plots were generated with log2 fold change on the x-axis and –log10(adjusted p-value) on the y-axis.

### 2.8 Cell-cell communication and transcription factor activity analysis

Cell-cell communication analysis: Intercellular signaling was inferred using LIANA (LIgand-receptor ANAlysis; version 0.1.12), which integrates five complementary scoring methods, including CellPhoneDB, NATMI, Connectome, logFC, and SingleCellSignalR, with results aggregated using rank_aggregate. Analyses were performed based on the consensus ligand-receptor resource (expr_prop = 0.1, n_perms = 100, seed = 42). Significant interactions were defined as those with both magnitude_rank < 0.05 and specificity_rank < 0.05. Fibroblast-centered communication was further characterized by restricting the source cell types to fibroblasts and myCAFs.

Transcription factor (TF) activity inference: TF activity was inferred using the Univariate Linear Model (ULM) method implemented in decoupler (version 2.1.6), with the DoRothEA gene regulatory network as the prior knowledge resource (confidence levels A-C; 32,286 interactions and 429 TFs). ULM activity scores were calculated for each cell and then averaged across fibroblast subtypes. To identify MMP2-associated transcriptional regulators, Spearman correlation analysis was performed between the activity scores of each TF and MMP2 expression across all fibroblast cells, restricted to TFs annotated as direct regulators of MMP2 in the DoRothEA network.

Signaling network visualization: A directed graph of fibroblast-target cell interactions was constructed using NetworkX (version 3.1). Node size was scaled according to the total interaction weight, and edge width represented the number of significant ligand-receptor pairs. The computationally inferred MMP2-PECAM1 pair from fibroblasts to endothelial cells was displayed separately in the network visualization.

### 2.9 Immunohistochemistry (IHC) staining

Tissue microarray sections were deparaffinized, rehydrated, and subjected to antigen retrieval in citrate buffer (pH 6.0). Primary antibodies against MMP2 (1:200, ab97779, Abcam), COL-1 (1:1000, HA722517, HUABIO, China) and FBXW7 (0.5ug/ml, ab109617, Abcam) were applied overnight at 4 °C, followed by incubation with horseradish peroxidase-conjugated secondary antibodies. Diaminobenzidine (DAB) was used for color development, and sections were counterstained with hematoxylin.

### 2.10 External validation of MMP2 using the GSE150290 and TCGA-STAD cohorts

To independently validate our in-house single-cell findings, we retrieved the publicly available GSE150290 scRNA-seq dataset from GEO, comprising paired normal gastric mucosa and tumor tissues from 29 gastric cancer patients (52 libraries). Expression matrices were read into R (v4.x) and assembled into a merged Seurat (v5) object with per-cell annotation of sample, patient, and tissue type (Normal/Tumor). Cells were retained if nFeature_RNA ≥ 200 and mitochondrial fraction < 20%, normalized by LogNormalize (scale.factor = 10,000), and 2,000 highly variable genes were selected by variance stabilizing transformation. PCA was run on the 2,000 HVGs, and the top 30 PCs (per elbow plot) were used to build a KNN graph (FindNeighbors) followed by Louvain clustering (FindClusters, resolution = 0.5) and UMAP visualization. Six major cell types were annotated by scoring canonical marker sets—Epithelial (EPCAM, KRT18, KRT19, CDH1), Fibroblasts (COL1A1, DCN, FAP, LUM), T cells (CD3D, CD3E, CD2, IL7R), B cells (CD79A, MS4A1, CD19, BANK1), Myeloid (LYZ, S100A8, S100A9, FCN1), and Endothelial (PECAM1, VWF, CDH5, CLDN5)—assigning each cluster the highest-scoring label, with validation by FeaturePlot, VlnPlot, and Heatmap. Fibroblasts were then re-extracted and classified into myCAFs (MMP2, COL1A1, PDGFRA, FAP, FN1, POSTN) and UFs (DCN, LYVE1), visualized by UMAP and DotPlot. For bulk-level validation, TCGA-STAD transcriptomic data (415 tumors, 34 normals) were used to compare MMP2 expression (TPM). MMP2 protein was assessed by immunohistochemistry (DAB chromogen) on normal and gastric cancer paraffin sections. IHC staining intensity was scored by two independent pathologists using Image-Pro Plus 6.0. Kaplan-Meier survival curves were plotted, and differences were evaluated by the log-rank test (*P* < 0.05). Between-group comparisons used the Mann-Whitney U test (two-sided), correlations used Spearman’s ρ, and survival differences used the Log-rank test; α = 0.05, p < 0.001 denoted ***.

### 2.11 Cell transfection and molecular assays

Plasmid construction and transfection: Full-length human MMP2 cDNA was cloned into the pcDNA3.1 vector for overexpression (OE-MMP2), while FBXW7 shRNA (KD-FBXW7) or control shRNA (NC) was cloned into the pLKO.1 vector. Transfections were performed using Lipofectamine 3000 (Invitrogen) according to the manufacturer’s instructions.

qRT-PCR: Total RNA was extracted using TRIzol reagent (Invitrogen), and cDNA was synthesized with the PrimeScript RT Reagent Kit (TaKaRa). qPCR was performed on a CFX96 Real-Time System (Bio-Rad) using SYBR Green Master Mix (TaKaRa). Primer sequences were listed as follows: MMP2 forward 5’-CCTGCTGCTCTACCTGATGC-3’, reverse 5’-GCTGCTGCTGCTGCTGCTG-3’; GAPDH forward 5’-GAAGGTGAAGGTCGGAGTC-3’, reverse 5’-GAAGATGGTGATGGGATTTC-3’).

Western blot: Cell lysates were separated by 10% SDS-PAGE and transferred to PVDF membranes. Primary antibodies against MMP2 (1:500), FBXW7 (1 µg/m, ab109617, Abcam), and GAPDH (1:5000, ab8245, Abcam) were used, followed by HRP-conjugated secondary antibodies. Protein bands were detected with an ECL kit (Millipore).

### 2.12 Functional phenotype assays

Proliferation assay: Cells (5×10³/well) were seeded in 96-well plates, and cell viability was measured using a CCK-8 kit (Dojindo) at 0, 24, 48, 72 and 96 h.

Wound healing assay: Confluent cell monolayers were scratched with a 200 μL pipette tip, and images were captured at 0h and 72 h using an inverted microscope (Nikon).

Transwell invasion assay: Cells (1×10^4^) were seeded in the upper chamber of Transwell inserts (8 μm pore size, Corning) coated with Matrigel (1:8 dilution, BD Biosciences). Medium containing 10% FBS was added to the lower chamber. After 24 h, invasive cells were fixed, stained with crystal violet, and counted in five random fields.

### 2.13 Protein interaction validation

**Immunofluorescence (IF):** Cells were fixed with 4% paraformaldehyde, permeabilized using 0.1% Triton X-100, and blocked with 5% BSA. Primary antibodies against FBXW7 (1:200), MMP2 (1:200) were incubated overnight, followed by incubation with Alexa Fluor 488-conjugated (green) and Alexa Fluor 594-conjugated (red) secondary antibodies (Invitrogen). Nuclei were counterstained with DAPI, and images were acquired using a confocal microscope (Leica).

Tyramide signal amplification (TSA): TSA signal amplification was performed per the commercial kit manual on fixed cell or formalin-fixed paraffin-embedded tissue sections. Samples underwent antigen retrieval, followed by 30 min room-temperature blocking with kit-supplied blocking buffer to suppress non-specific binding. Primary antibody (MMP2): incubation proceeded overnight at 4 °C; slides were washed 3 × 5 min with kit wash buffer before incubation with HRP-conjugated secondary antibody for 50 min at ambient temperature. Fresh fluorescent tyramide working solution was prepared by diluting tyramide stock in amplification buffer, applied to slides and incubated 15 min in the dark to enable HRP-catalyzed covalent tyramide deposition near target epitopes. The reaction was terminated with stop reagent, followed by three buffer washes. Nuclear counterstaining with DAPI (1 μg mL-1, 5 min) was conducted prior to antifade mounting. Fluorescent signals were imaged via confocal microscopy with matched fluorophore filter sets, The primary antibodies and their dilution ratios were as follows: MMP2 (1:1000, HUABIO, China), FAP (1:1000, HUABIO, China), (CK18, 1:1000, HUABIO, China).

Co-immunoprecipitation (Co-IP): Cells transfected with FBXW7-Flag plasmids were lysed, and the resulting cell extracts were incubated with anti-Flag M2 magnetic beads (Sigma) overnight at 4 °C. The immunocomplexes were then washed, eluted, and subjected to Western blot analysis using anti-MMP2 and anti-Flag antibodies.

Immunoprecipitation-mass spectrometry (LC-MS) analysis: LC-MS analysis was performed by Jingzhou Gene Technology (shanghai, China). Briefly, protein samples were separated by SDS-PAGE and subjected to overnight tryptic digestion. The resulting peptide mixtures were then desalted using a desalting column (19, 20). The digested peptides were analyzed with a Q Exactive mass spectrometer (Thermo Fisher Scientific, Massachusetts, USA). The fragment spectra was analyzed using the Proteome Discoverer 2. 2.

### 2.14 Ubiquitination assay

To assess FBXW7-mediated ubiquitination, HEK293T cells were co-transfected with HA-Ub (WT, K48, or K63) and either FBXW7-Flag or an empty vector (EV) control using polyethylenimine (PEI). To prevent proteasomal degradation and enrich ubiquitinated proteins, cells were treated with 20 μM MG132 for 4 h prior to lysis. Subsequently, the ubiquitination levels of MMP2 were evaluated via co-immunoprecipitation (Co-IP) using an anti-HA antibody.

### 2.15 Ethics statement

All methods were carried out in accordance with relevant guidelines and regulations. This study is reported in accordance with ARRIVE guidelines (https://arriveguidelines.org).

Clinical sample usage was approved by the Ethics Committee of the First Affiliated Hospital of Chongqing Medical University (Approval No: K2023-100). Animal experiments were conducted in accordance with the institutional guidelines and approved by the Animal Ethics Committee of Enshi Tujia and Miao Autonomous Prefecture Central Hospital (Approval No: W202410002).

### 2.16 Statistical analysis

All statistical analyses were performed using R (version 4.2.3), Python (3.10.19), and GraphPad Prism 9.0. Data are presented as the mean ± standard deviation (SD). Comparisons between groups were conducted using Student’s t-test for two groups or one-way ANOVA followed by Dunnett’s multiple comparisons test for multiple groups. Correlations between MMP2 and PMS2 expression were assessed using Pearson’s correlation coefficient. A value of *P* < 0.05 was considered statistically significant (*P < 0.05, **P < 0.01, ***P < 0.001, ****P < 0.0001).

## 3 Results

### 3.1 Single-cell transcriptomic landscape identifies fibroblast enrichment and MMP2 upregulation in gastric cancer

**Figure 1:**
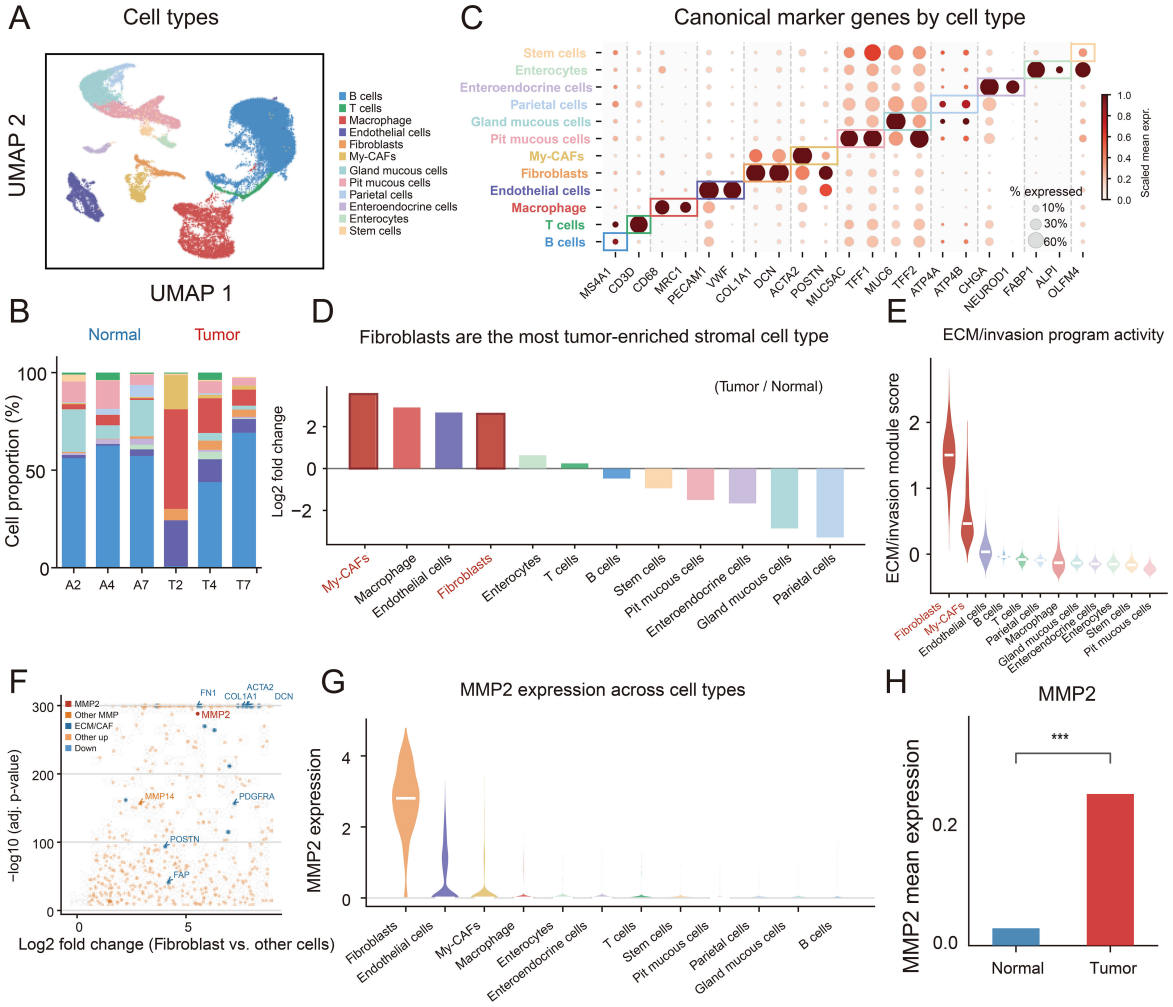
Single-cell transcriptomic landscape identifies fibroblast enrichment and MMP2 upregulation in gastric cancer. **(A)** UMAP visualization of 12 major cell populations identified from integrated scRNA-seq data of gastric cancer (Tumor) and adjacent normal (Normal) tissues. **(B)** Stacked bar charts showing cell-type proportional composition per sample (A2, A4, A7: normal; T2, T4, T7: tumor). **(C)** Dot plot of canonical marker genes for each cell type. Dot size: percentage of cells expressing the gene; color intensity: scaled mean expression. **(D)** Bar chart of log2 fold change in cell-type abundance (Tumor vs. Normal), demonstrating that myCAFs and Fibroblasts are the most tumor-enriched stromal cell types. **(E)** Violin plots of ECM/invasion program module scores across all 12 cell types; Fibroblasts and myCAFs exhibit the highest scores. **(F)** Volcano plot of Fibroblasts versus all other cell types, highlighting MMP2 as a top upregulated gene. **(G)** Violin plots of MMP2 expression across all 12 cell populations; expression is highest in Fibroblasts and myCAFs. **(H)** Bar chart of mean MMP2 expression in normal versus tumor samples. ***P < 0.001.

To systematically characterize the cellular composition of the gastric TME, we performed scRNA-seq on paired tumor and adjacent normal tissues from three gastric cancer patients (six samples: A2, A4, A7 [normal]; T2, T4, T7 [tumor]). After quality control, normalization, and Harmony-based batch correction, unsupervised Leiden clustering identified 12 major cell populations visualized by UMAP (**Figure 1A**). These comprised immune cells (B cells, T cells, Macrophages), stromal components (Fibroblasts, myCAFs, Endothelial cells), and epithelial populations (Pit mucous cells, Gland mucous cells, Parietal cells, Enteroendocrine cells, Enterocytes, Stem cells), each validated by canonical marker gene expression (**Figure 1C**). Cell proportion analysis across samples revealed pronounced compositional differences between tumor and normal tissue (**Figure 1B**).

Among all stromal cell types, fibroblast populations (Fibroblasts and myCAFs) showed the greatest tumor enrichment, with log2 fold changes exceeding those of all other cell types (**Figure 1D**). To quantify the functional consequence of this stromal expansion, we computed an ECM/invasion program module score per cell. Fibroblasts and myCAFs exhibited significantly higher ECM/invasion activity than all immune and epithelial cell types (**Figure 1E**), implicating them as the principal drivers of matrix remodeling in the gastric cancer TME. Differential expression analysis comparing fibroblasts against all other cells identified MMP2 as one of the most significantly upregulated genes (**Figure 1F**). MMP2 expression was highest in Fibroblasts and myCAFs across all 12 cell populations (**Figure 1G**), and its mean expression was significantly elevated in tumor versus adjacent normal tissue (**Figure 1H**, *P* < 0.001). These analyses nominate MMP2, preferentially expressed within the expanded fibroblast compartment, as a key mediator of the invasive phenotype in gastric cancer TME.

### 3.2 MMP2-expressing universal fibroblasts represent a transitional CAF state with ECM remodeling programs

**Figure 2:**
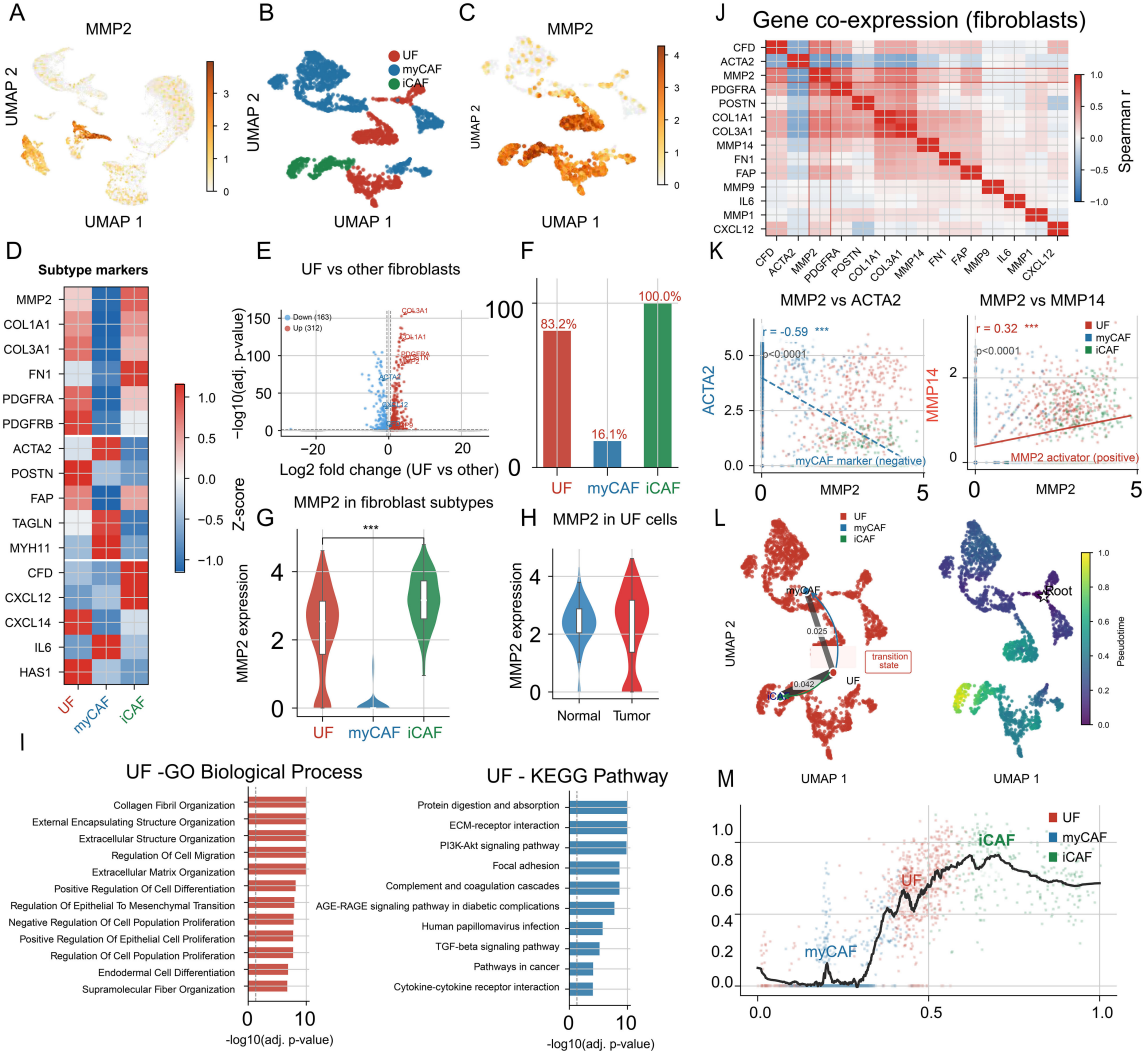
MMP2-expressing universal fibroblasts represent a transitional CAF state with ECM remodeling programs. **(A)** UMAP of all cells colored by MMP2 expression level. **(B)** UMAP of three fibroblast subtypes: Universal Fibroblasts (UF, teal), myofibroblastic (myCAFs, red), and inflammatory CAFs (iCAF, orange). **(C)** UMAP of the fibroblast subset colored by MMP2 expression, showing concentration in UF and myCAF regions. **(D)** Heatmap of subtype-specific marker gene expression across UF, myCAF, and iCAF. **(E)** Volcano plot of UF versus other fibroblast subtypes, with MMP2 as the top upregulated gene. **(F)** Percentage of MMP2-positive cells per fibroblast subtype: UF (83.3%), myCAF (100.0%), iCAF (16.1%). **(G)** Violin plot of MMP2 expression across three fibroblast subtypes. ***P < 0.001. **(H)** Violin plot of MMP2 expression in UF cells from normal versus tumor tissue. ***P < 0.001. **(I)** GO Biological Process (left) and KEGG pathway (right) enrichment of UF-specific upregulated genes. **(J)** Spearman co-expression heatmap of key fibroblast genes across all fibroblast cells. **(K)** Scatter plots of MMP2 vs. ACTA2 (left; r=−0.59, *P* < 0.001) and MMP2 vs. MMP14 (right; r=+0.32, *P* < 0.001), colored by fibroblast subtype. **(L)** PAGA trajectory overlaid on UMAP, depicting developmental connectivity between UF, myCAF, and iCAF subtypes with diffusion pseudotime coloring. **(M)** Kernel density curves of diffusion pseudotime per fibroblast subtype, confirming UF cells occupy early pseudotime and represent a transitional state.

To resolve the heterogeneity within the expanded fibroblast compartment, we sub-clustered fibroblast and My-CAF cells and identified three functionally distinct subtypes: Universal Fibroblasts (UF), myCAF, and inflammatory CAFs (iCAF) (**Figure 2B**). MMP2 expression was spatially concentrated in the UF and myCAF regions of the fibroblast UMAP landscape (**Figures 2A, C**). Quantification revealed that 83.3% of UF cells and 100% of myCAF cells expressed detectable MMP2, compared to only 16.1% of iCAF cells (**Figure 2F**), with MMP2 being the top differentially expressed gene in UF versus other fibroblast subtypes (**Figure 2E**). Marker analysis confirmed that UF cells co-express MMP2, COL1A1, FN1, and PDGFRA; myCAFs are defined by contractile markers ACTA2, POSTN, and MYH11; and iCAFs express inflammatory mediators CXCL12, CXCL14, and IL6 (**Figure 2D**). MMP2 expression in UF cells was significantly higher in tumor versus adjacent normal tissue (**Figure 2H**, *P* < 0.001), confirming tumor-context upregulation. Gene co-expression analysis revealed strong positive correlations between MMP2 and ECM genes COL1A1, FN1, and COL3A1, while MMP2 was negatively correlated with the myCAF marker ACTA2 (Spearman r=−0.59, *P* < 0.001) and positively correlated with the MMP2-activating enzyme MMP14 (r=+0.32, *P* < 0.001) (**Figures 2J, K**).

Functional enrichment analysis of UF-specific upregulated genes revealed significant enrichment in GO Biological Process terms related to collagen fibril organization, extracellular matrix organization, and regulation of cell migration, as well as KEGG pathways including ECM-receptor interaction, PI3K-Akt signaling, focal adhesion, and TGF-beta signaling (**Figure 2I**). PAGA trajectory analysis showed that UF cells occupy the root position of the fibroblast developmental continuum, with bidirectional connectivity to both myCAF (connectivity=0.025) and iCAF (connectivity=0.042) (**Figure 2L**). Diffusion pseudotime analysis confirmed that UF cells predominate at early pseudotime while myCAFs accumulate at late pseudotime, supporting a model in which UF represents a transitional intermediate capable of differentiating toward either a contractile myCAF or inflammatory iCAF fate (**Figure 2M**). Together, these data establish UF cells as the primary MMP2-producing subpopulation of the gastric cancer stroma, with MMP2 expression representing a functional hallmark of this transitional state.

### 3.3 Fibroblast-centered intercellular communication and transcriptional control of MMP2 in the gastric cancer microenvironment

**Figure 3:**
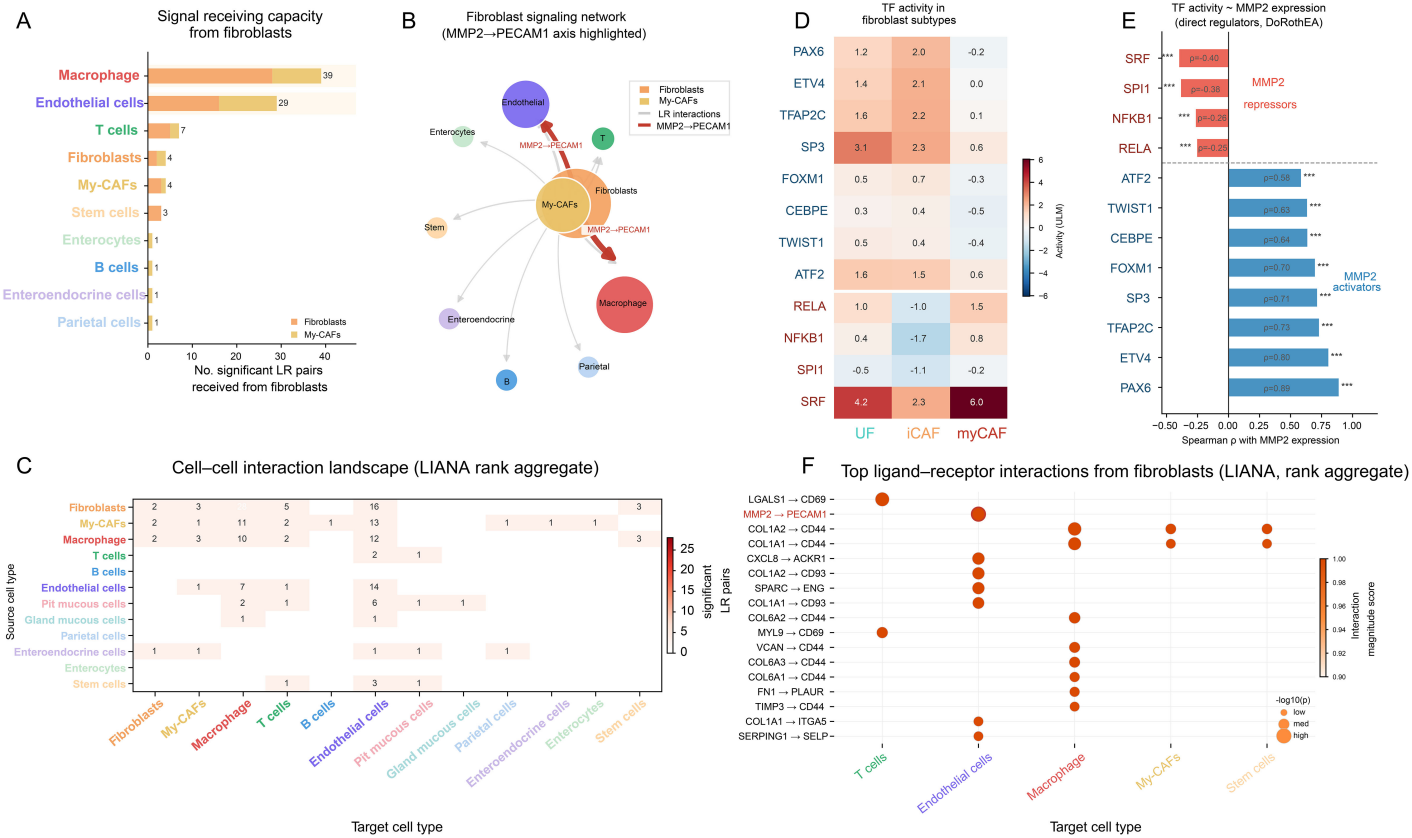
Fibroblast-centered intercellular communication and transcriptional control of MMP2 in the gastric cancer microenvironment. **(A)** Horizontal bar chart of the number of significant LR pairs received by each cell type from fibroblast sources (Fibroblasts, orange; myCAFs, yellow). Macrophages and Endothelial cells are the dominant receivers. **(B)** Directed network graph of fibroblast signaling. Node size is proportional to total interaction strength; edge width reflects LR pair count. The MMP2→PECAM1 axis (Fibroblasts→Endothelial cells) is highlighted in red. **(C)** Heatmap of scaled cell–cell interaction scores (LIANA rank_aggregate) across all cell-type pairs; color intensity represents interaction magnitude. **(D)** Heatmap of transcription factor activity (DoRothEA/ULM scores) across UF, iCAF, and myCAF subtypes, revealing SP3, ETV4, TFAP2C, and FOXM1 as UF-enriched TFs and SRF as the dominant myCAF TF. **(E)** Horizontal bar chart of Spearman correlation (ρ) between TF activity and MMP2 expression, restricted to direct DoRothEA-annotated MMP2 regulators. Blue bars: positive correlates (MMP2 activators); red bars: negative correlates (SRF, NFKB1, RELA). **(F)** Bubble plot of top ligand–receptor pairs originating from fibroblasts (LIANA rank_aggregate). Bubble size: significance score (–log10 adjusted p-value); color: interaction magnitude rank.

LIANA analysis identified predicted interactions between fibroblasts and multiple cell populations (**Figure 3C**). Macrophages and endothelial cells received 39 and 29 significant predicted ligand-receptor pairs, respectively (**Figure 3A**). The MMP2-PECAM1 pair was among the highly ranked fibroblast-to-endothelial predictions (**Figures 3B, F**).

To identify the transcriptional drivers of MMP2 in UF cells, we inferred transcription factor activity using the DoRothEA/ULM method. TF activity heatmap analysis showed that ETV4, SP3, TFAP2C, FOXM1, TWIST1, and ATF2 were most active in UF relative to myCAF (**Figure 3D**). Spearman correlation of TF activity scores against MMP2 expression across all fibroblast cells—restricted to DoRothEA-annotated direct MMP2 regulators—revealed that ETV4 (ρ=+0.80), SP3 (ρ=+0.71), FOXM1 (ρ=+0.70), TWIST1 (ρ=+0.63), and ATF2 (ρ=+0.58) were the strongest positive correlates of MMP2 expression (**Figure 3E**). Conversely, SRF (ρ=−0.40), which drives the contractile myCAF phenotype via actin cytoskeleton remodeling, showed a strong negative correlation with MMP2, consistent with MMP2 expression being suppressed upon terminal myCAF differentiation. These findings establish ETV4 and SP3 as the primary transcriptional activators of MMP2 in the UF subpopulation, operating within a signaling network that channels stromal MMP2 toward the MMP2→PECAM1 vascular axis.

### 3.4 MMP2 is markedly upregulated in gastric cancer and is associated with poor prognosis.

**Figure 4:**
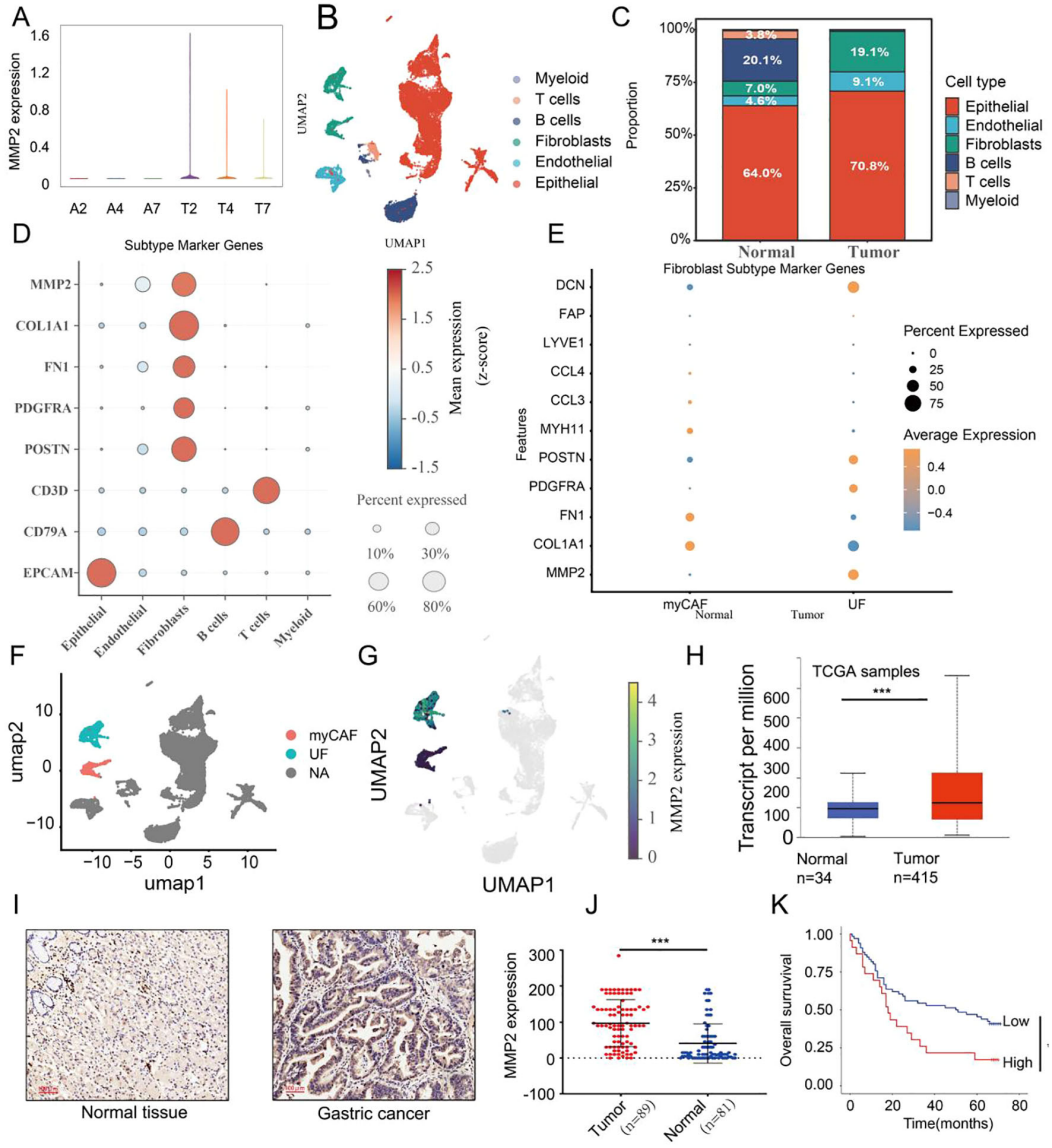
MMP2 is significantly upregulated in gastric cancer, originates primarily from myCAF fibroblasts, and correlates with poor prognosis. **Figure 4** Multi-omics integrative analysis of MMP2 in gastric cancer. **(A)** Violin plot of MMP2 expression in paired normal (A2, A4, A7) and tumor (T2, T4, T7) tissues. **(B)** UMAP of six major cell types from the GSE150290 dataset. **(C)** Bar chart of cell-type proportions in normal versus tumor tissues. **(D)** Dot plot of MMP2 and ECM genes across six cell types; dot size = % positive cells, color = scaled mean expression. **(E)** Marker gene expression comparing myCAF and UF subtypes (orange = high, blue = low). **(F)** UMAP of fibroblast subtypes: myCAFs (red), UFs (cyan), unassigned (grey). **(G)** UMAP of all cells colored by MMP2 expression (yellow = high, purple = low). **(H)** Box plot of MMP2 TPM in TCGA-STAD (Tumor n = 415, Normal n = 34); ***p < 0.001, Wilcoxon test. **(I)** Immunohistochemistry was used to detect the expression of MMP2 in normal tissues and gastric cancer tissues (scale scale = 100 μm) in gastric cancer tissue microarray. **(J)** The expression of MMP2 in 89 gastric cancer tissues and 81 adjacent normal tissues by utilizing IHC in human gastric cancer tissue chip (HColA180Su01) (Tumor n = 89, red; Normal n = 81, blue); ***p < 0.001. **(K)** Kaplan-Meier survival curves for MMP2 high (red) versus low (blue) expression; Log-rank test, p < 0.05.

MMP2 is significantly upregulated in gastric cancer tissues. Comparison of MMP2 expression in paired normal (A2, A4, A7) and tumor (T2, T4, T7) samples showed marked upregulation in tumor samples—highest in T2 (~1.6), then T4 (~0.9) and T7-while normal samples remained near baseline (**Figure 4A**), indicating that MMP2 is induced in the gastric cancer TME. The tumor microenvironment undergoes significant cellular compositional remodeling. UMAP classified high-quality single cells into six major cell types-Epithelial, Endothelial, Fibroblasts, B cells, T cells, and Myeloid-each forming distinct clusters (**Figure 4B**). Proportional comparison revealed that fibroblasts were significantly enriched in tumor tissues, myeloid cells showed an expansion trend, and epithelial cells decreased (**Figure 4C**), consistent with a desmoplastic stromal response. Fibroblasts are the principal cellular source of MMP2 and ECM remodeling genes. A DotPlot across six cell types showed MMP2 was specifically high in fibroblasts (mean 1.26, 57.4% positive) and very low in the other five types (<0.32); COL1A1 (2.58, 86.1%), FN1 (1.07, 47.7%), POSTN (1.73, 61.1%), and PDGFRA (0.85, 42.4%) were likewise fibroblast-specific. Canonical markers confirmed annotation (CD3D in T cells 2.13; CD79A in B cells 2.03; EPCAM in epithelial cells 1.58) (**Figure 4D**). Fibroblasts comprise two functional subtypes: myCAF and UF. myCAFs overexpressed ECM degradation/remodeling genes (FAP, PDGFRα, COL1A1, MMP2), whereas UFs relatively overexpressed DCN and LYVE1 (**Figure 4E**). UMAP showed myCAFs (red) and UFs (cyan) formed partially separated distributions, indicating distinguishable transcriptomic features (**Figure 4F**). MMP2 exhibits spatially heterogeneous expression in the tumor microenvironment. Overlaying the MMP2 expression gradient on the whole-cell UMAP showed MMP2-high cells (yellow, 2–4) concentrated in fibroblast/myCAF regions, while most background cells were MMP2-low (purple), confirming at single-cell resolution that MMP2 is driven by the myCAF subpopulation rather than uniformly expressed (**Figure 4G**). The TCGA cohort externally validates MMP2 upregulation. In TCGA-STAD, median MMP2 TPM was significantly higher in tumors (n = 415) than normal tissues (n = 34) (***p < 0.001, Wilcoxon rank-sum test), validating MMP2 upregulation in a large independent cohort (**Figure 4H** and **Supplementary Table 2**). Immunohistochemical results of gastric cancer tissue microarray showed that MMP2 staining was weak (light brown) in normal gastric tissue, but was extensively and strongly positive (dark brown) in tumor stroma (scale = 100 μm), confirming that MMP2 protein levels were upregulated in the interstitial fibroblast region (**Figure 4I**). Further statistical analysis showed that the protein level of MMP2 was significantly up-regulated in human gastric cancer tissues, and the difference was statistically significant (**Figure 4J** and **Supplementary Table 3**). Kaplan-Meier analysis showed that high-expression patients (red) had significantly shorter median and long-term overall survival than low-expression patients (blue) (Log-rank test, p < 0.05), supporting MMP2 as an independent prognostic biomarker (**Figure 4K**). Next, the results of COX multivariate regression analysis confirmed that the expression level of MMP2 and N stage were significantly correlated with the prognosis of gastric cancer (**Table 1**, P<0.05), which could be used as an independent prognostic factor for gastric cancer patients. Taken together, our experiments demonstrate the biological relevance of MMP2 in gastric cancer progression and support its potential utility as a prognostic biomarker.

**Table 1:** Univariate and multivariate analyses of the factors correlated with overall survival of cancer patients.

variables	Univariate analysis			Multivariate analysis		
	HR	95%CI	p value	HR	95%CI	p value
MMP2	2.888	1.618-5.154	**0.000335**	1.93	1.04-3.59	**0.0379**
expression						
Sex	0.767	0.441-1.333	0.347			
Age	1.108	0.649-1.89	0.708			
Tumor size	1.185	0.688-2.04	0.54			
T stage	2.227	1.182-4.197	**0.0133**	1.55	0.77-3.1	0.218
N stage	3.733	1.592-8.754	**0.00245**	2.54	0.93-6.94	**0.068**
TNM stage	2.867	1.57-5.234	**0.000604**	1.48	0.71-3.08	0.298
Grade stage	0.899	0.489-1.655	0.733			

Tumor Staging Guidelines: AJCC Cancer Staging Manual (8th). T-Stage, Tumor invasion stage; N stage, Lymph nodes metastasis stage; TNM stage, tumor-node-metastasis stage. P < 0.05:

statistically significant. Bold values indicate statistical significance (P < 0.05).

Given the pervasive cytoplasmic localization of MMP2 in tumor epithelial cells observed in our IHC analysis, and considering the established role of MMP2 in promoting invasive phenotypes, we next sought to dissect the cell-autonomous functional contributions of MMP2 in gastric cancer cells.

### 3.5 FBXW7 inhibits MMP2-mediated gastric cancer cell migration and invasion

**Figure 5:**
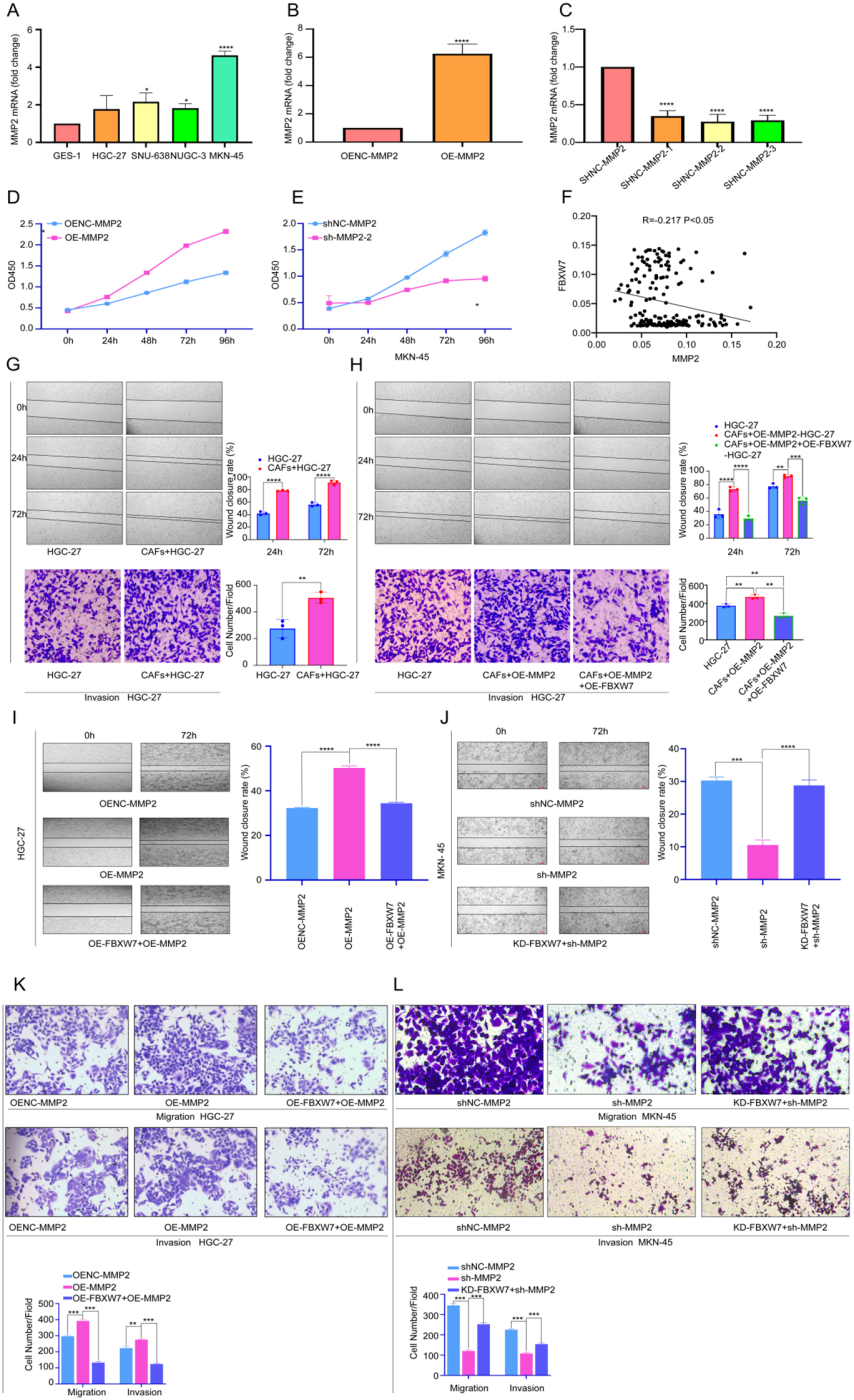
FBXW7 Inhibits MMP2-Mediated Gastric Cancer Cell Migration and Invasion. All experiments were performed in triplicate. Data are presented as the mean ± standard deviation (SD). Statistical analyses were performed using either one-way or two-way ANOVA, as appropriate. Significance levels are denoted as follows: *P < 0.05, **P < 0.01, ***P < 0.001, and ****P < 0.0001. **(A)** MMP2 mRNA expression in normal gastric epithelial cells (GES-1) and gastric cancer cell lines (HGC-27, SNU-638, NUGC-3, MKN-45) analyzed by qRT-PCR. **(B)** MMP2 mRNA levels in MMP2-overexpressing (OE-MMP2) and control (OENC-MMP2) HGC-27 cells. **(C)** MMP2 mRNA expression in MMP2-silenced (sh-MMP2-1/-2/-3) and control (shNC-MMP2) MKN-45 cells. **(D)** The viability of HGC-27 cell line of OE-MMP2 and OENC-MMP2 assessed by CCK-8 assay over 96h. **(E)** The viability of MKN-45 cell line of sh-MMP2–2 and shNC-MMP2 evaluated by CCK-8 assay over 96h. **(F)** Correlation analysis of FBXW7 and MMP2 in the immunohistochemical results of gastric cancer tissue microarray in this project. **(G)** Wound healing and Transwell experiments in HGC-27 and CAFs + HGC-27 co-culture systems, bar graphs represent their analysis results. **(H)** Wound healing and Transwell experiments in HGC-27, CAFs + OE-MMP2 - HGC-27 and CAFs + OE-MMP2+OE-FBXW7-HGC-27 co-culture systems, bar graphs represent their analysis results. **(I)** Wound healing assay of OENC-MMP2, OE-MMP2, and OE-FBXW7+OE-MMP2 - HGC-27 cells, bar graphs represent their analysis results. **(J)** Wound healing assay of shNC-MMP2, sh-MMP2-2, and KD-FBXW7+sh-MMP2-2 - MKN-45 cells, bar graphs represent their analysis results. **(K)** Transwell migration and invasion assays of OENC-MMP2, OE-MMP2, and OE-FBXW7+OE-MMP2 - HGC-27 cells, bar graphs represent their analysis results. **(L)** Transwell migration assay of shNC-MMP2, sh-MMP2-2, and KD-FBXW7+sh-MMP2-2 - MKN-45 cells, bar graphs represent their analysis results.

To systematically elucidate the oncogenic role of MMP2 in gastric carcinogenesis, we initially quantified its mRNA expression in the normal human gastric mucosal epithelial cell line (GES-1) and four gastric cancer cell lines (HGC-27, SNU-638, NUGC-3, and MKN-45) using quantitative reverse transcription PCR (qRT-PCR). Consistent with the elevated MMP2 expression observed in gastric cancer tissues, significantly higher mRNA levels were detected in gastric cancer cell lines, particularly SNU-638 and MKN-45, compared to GES-1(**Figure 5A**). To further investigate the biological function of MMP2, we constructed plasmids for both overexpression and knockdown. The overexpression efficiency was validated in HGC-27 cells (**Figure 5B**), while the knockdown efficiency was confirmed in MKN-45 cells (**Figure 5C**), demonstrating that both constructs met the experimental requirements. Subsequent functional assays revealed that MMP2 overexpression in HGC-27 cells significantly enhanced cell viability (**Figure 5D**), whereas MMP2 knockdown in MKN-45 cells significantly inhibited cell viability (**Figure 5E**). These findings suggest that MMP2 plays an important role in promoting the proliferative capacity of gastric cancer cells.

In our previous study, our team discovered that FBXW7 can regulate proteins such as VDAC3 (14) and BGN (15)through the ubiquitination pathway. And we identified an interaction between FBXW7 and MMP2 in HGC-27 cells through co-immunoprecipitation and mass spectrometry analyses (**Supplementary Table 4**). Given that MMP2 is a well-established mediator of tumor metastasis, we hypothesized that FBXW7 might play a critical role in MMP2-mediated tumor metastasis. Therefore, we examined the correlation between FBXW7 and MMP2 in the gastric cancer tissue chip. The results showed that FBXW7 and MMP2 exhibited a negative correlation (*P* < 0.05) (**Figure 5F**). In order to confirm the effect of CAFs on the migration and invasion of gastric cancer cells and evaluate the regulatory effects of MMP2 and FBXW7 on tumor cell migration and invasion, based on single-cell sequencing analysis, it was found that fibroblasts were the main source of MMP2. Wound healing and Transwell assays using CAFs + HGC-27 gastric cancer cell co-culture system showed that CAFs could promote the migration and invasion of gastric cancer cells, and FBXW7-MMP2 was also confirmed to regulate the migration and invasion of gastric cancer cells (**Figures 5G, H**). Then, to test the FBXW7-MMP2 axis in gastric cancer cell migration and invasion, we conducted wound healing assays and the results revealed that MMP2 overexpression (OE-MMP2) in HGC-27 cells significantly increased the wound closure rate compared to the control group, indicating enhanced migratory capacity (**Figure 5I**). Conversely, MMP2 knockdown (sh-MMP2) in MKN-45 cells drastically inhibited wound healing (**Figure 5J**). Crucially, the pro-migratory effect induced by MMP2 overexpression (OE-MMP2) was effectively rescued by the concurrent overexpression of FBXW7 (OE-FBXW7+OE-MMP2), as evidenced by the accelerated wound closure (**Figures 5I, J**). Similarly, in MKN-45 cells, the suppression of migration caused by MMP2 knockdown (sh-MMP2) was reversed by the additional knockdown of MMP2 (KD-FBXW7+sh-MMP2) (**Figure 5K**). Meanwhile, Transwell invasion assays further confirmed these findings. Overexpression of MMP2 (OE-MMP2) significantly increased the number of migrated and invaded HGC-27 cells, while the combination of OE-FBXW7 and OE-MMP2 resulted in an attenuated increase (**Figure 5L**). In contrast, the knockdown of MMP2 (sh-MMP2) in MKN-45 cells significantly reduced the number of migrated and invaded cells. Notably, the inhibitory effect on invasion caused by MMP2 knockdown (sh-MMP2) was partially reversed by the simultaneous knockdown of FBXW7 (KD-FBXW7+sh-MMP2) (**Figures 5K, L**). Taken together, CAFs can promote the migration and invasion of gastric cancer cells, FBXW7 is negatively correlated with MMP2, and FBXW7 inhibits MMP2-mediated migration and invasion of gastric cancer cells.

### 3.6 Post-translational regulation of MMP2 by FBXW7-mediated proteasomal degradation

Our preliminary mass spectrometry data indicated an interaction between FBXW7 and MMP2. Cellular functional assays further demonstrated that FBXW7 overexpression attenuates MMP2-mediated tumor cell migration. Building on these findings, we sought to elucidate the underlying molecular mechanisms by which FBXW7 regulates MMP2.

**Figure 6:**
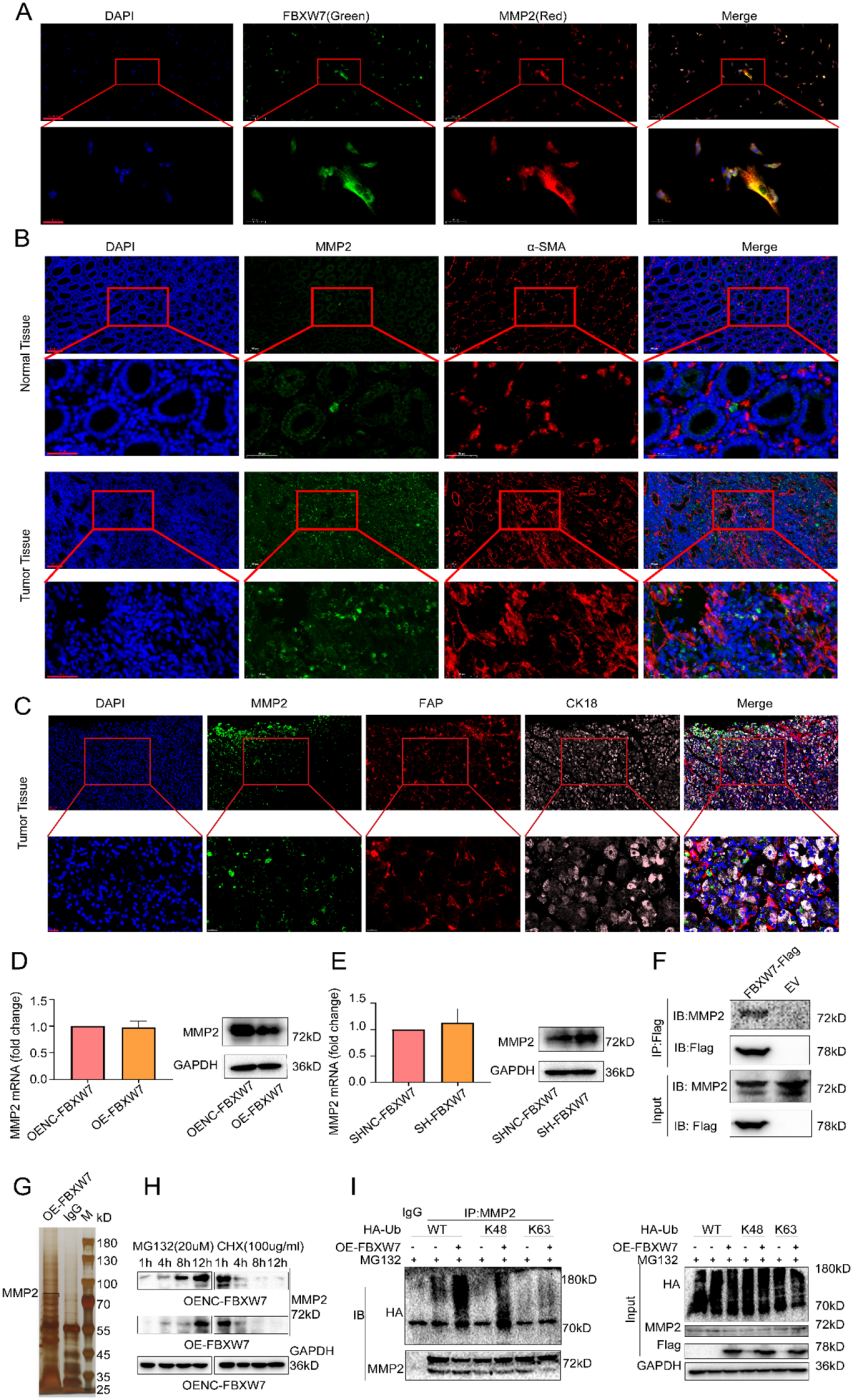
Post-translational Regulation of MMP2 by FBXW7-mediated Proteasomal Degradation. **(A)** Immunofluorescence staining of FBXW7 (green) and MMP2 (red) in gastric cancer cells, with DAPI (blue) for nuclear counterstaining. Merged images show their colocalization (yellow), indicating interaction (scale bar = 200 μm). **(B)** Immunofluorescence staining of FBXW7 (green) and α-SMA (red) in gastric cancer cells, with DAPI (blue) for nuclear counterstaining. Merged images show their colocalization (yellow), indicating interaction (scale bar = 200 μm). **(C)**: The co - localization of MMP2 (green), FAP (red), and CK18 (pink) proteins in human gastric cancer tissues were detected using an immune triple - labeling assay. **(D)** MMP2 mRNA expression in FBXW7-overexpressing (OE-FBXW7) and control (OENC-FBXW7) HGC-27 cells, with corresponding Western blot (WB) of MMP2 and GAPDH (loading control). **(E)** MMP2 mRNA expression in FBXW7-silenced (sh-FBXW7, not shown) and control (NC-FBXW7) HGC-27 cells, with WB validation (MMP2, GAPDH). **(F)** Co-immunoprecipitation (IP) in HGC-27 cells expressing FBXW7-flag, confirming FBXW7-MMP2 interaction. **(G)** Silver-stained pictures of Co-IP products obtained by silver-stained experiments. **(H)** WB analysis of MMP2 and GAPDH in HGC-27 cells treated with proteasome inhibitors (MG132, CHX) at indicated time points, showing MMP2 protein stability regulation (representative blots). **(I)** HEK293T cells were co-transfected with HA-tagged wild-type (WT), K48-only, or K63-only ubiquitin plasmids, in combination with FBXW7 overexpression plasmids or an empty vector (EV) control. Cell lysates were subjected to immunoprecipitation (IP) using an anti-MMP2 antibody, followed by immunoblotting (IB) with an anti-HA antibody to assess ubiquitination levels. Data are representative of three independent experiments.

To investigate the molecular regulation of MMP2, we first examined its interaction with FBXW7. Immunofluorescence staining showed the colocalization of FBXW7 and MMP2 in gastric cancer cells, as evidenced by merged yellow signals, suggesting a potential physical interaction (**Figure 6A**). Next, the expression of MMP2 in fibroblasts (marker: α-SMA) was confirmed by Immunofluorescence staining assay (**Figure 6B**). Next, the co-localization of MMP2, FAP and CK18 in human gastric cancer tissues was detected by multiple immunomulti-label staining. The results confirmed that secreted MMP2 was internalized from tumor cells, and there was extensive co-localization of MMP2, FAP and CK18 (**Figure 6C**). Quantitative reverse transcription PCR (qRT-PCR) analysis revealed no significant change in MMP2 mRNA levels upon FBXW7 overexpression compared to the control in HGC-27 cells, thereby excluding transcriptional regulation (**Figures 6C, D**). Co-immunoprecipitation assays further confirmed the interaction between FBXW7 and MMP2(**Figures 6E, F**). Subsequently, we assessed MMP2 protein stability using the proteasome inhibitor MG132 and the protein synthesis inhibitor cycloheximide (CHX). Western blot analysis showed a time-dependent increase in MMP2 levels in HGC-27 cells treated with these inhibitors, indicating that FBXW7 facilitates the proteasomal degradation of MMP2 (**Figure 6G**). Furthermore, we examined the ubiquitination level of MMP2 under FBXW7 overexpression, The results show that overexpression of FBXW7 significantly increased the level of poly-ubiquitinated MMP2 compared to the control group (**Figure 6H**). This result indicates that FBXW7 promotes the ubiquitination of MMP2, thereby facilitating its degradation via the proteasome pathway. To elucidate the specific molecular mechanism by which FBXW7 regulates MMP2 protein stability, we next investigated the type of ubiquitination involved in FBXW7-mediated MMP2 degradation. We performed co-immunoprecipitation assays in cells co-transfected with wild-type (WT), K48-only, or K63-only ubiquitin plasmids, alongside FBXW7 overexpression or an empty vector (EV) control. Western blot analysis revealed that FBXW7 overexpression significantly enhanced the overall ubiquitination level of MMP2. Subsequent linkage-specific analysis demonstrated that FBXW7 markedly increased the ubiquitination of MMP2 specifically via K48-linked polyubiquitin chains. In contrast, no significant change in MMP2 ubiquitination was observed under K63-linked conditions. Collectively, these results indicate that FBXW7 promotes the proteasomal degradation of MMP2 primarily by facilitating K48-linked polyubiquitination (**Figure 6I**). In summary, these findings demonstrate that FBXW7 interacts with MMP2 at the post-translational level, modulating its protein stability and consequently affecting MMP2’s oncogenic roles in gastric cancer.

### 3.7 FBXW7 suppresses MMP2-driven gastric tumor growth *in vivo*

**Figure 7:**
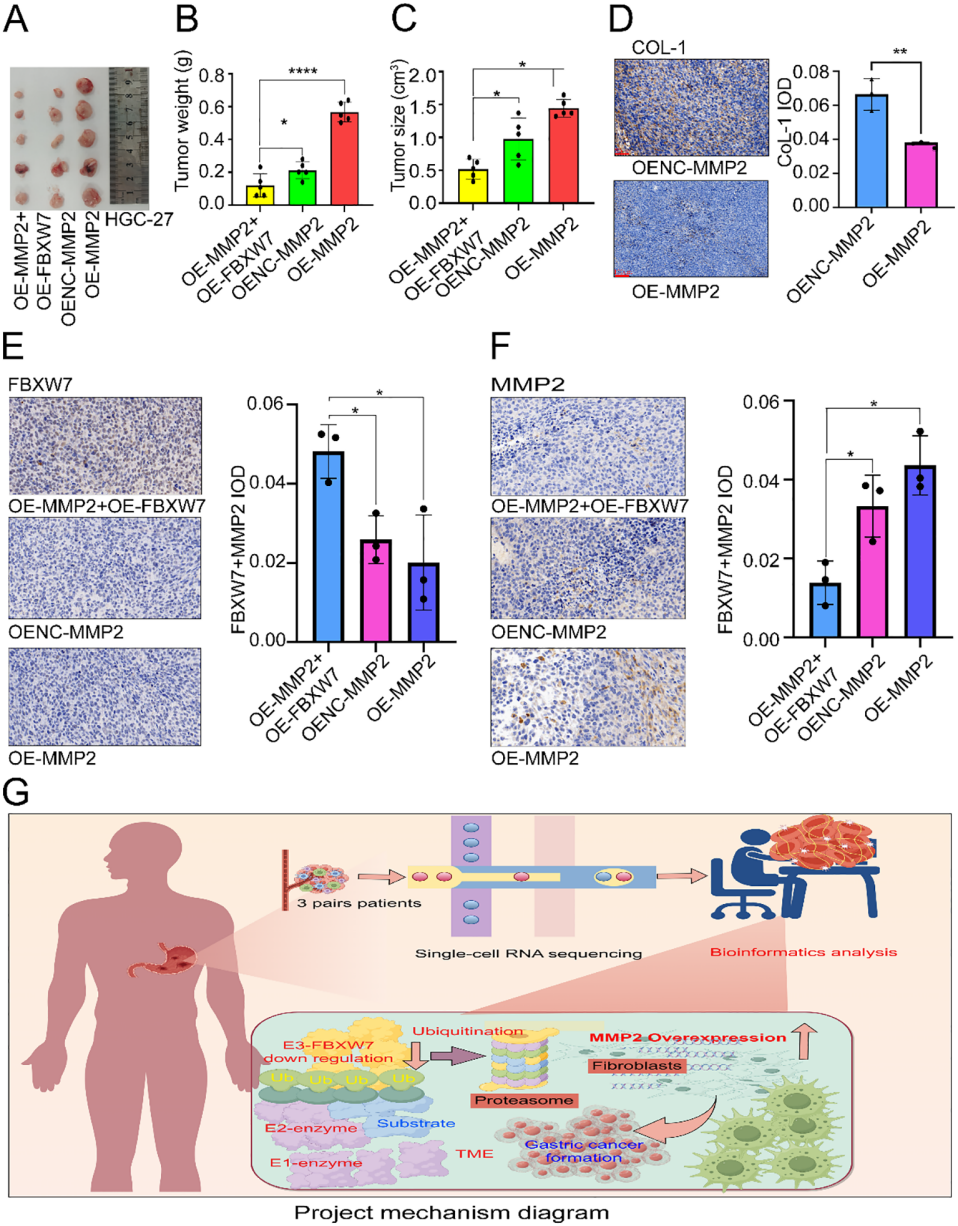
FBXW7 Suppresses MMP2-Driven Gastric Tumor Growth *In Vivo.* All *in vivo* experiments in triplicate; *in vivo* experiments with n = 5. Data are presented as the mean ± standard deviation (SD). Statistical analyses were performed using either one-way or two-way ANOVA, as appropriate. Significance levels are denoted as follows: *P < 0.05, **P < 0.01, ***P < 0.001, and ****P < 0.0001. **(A)** Representative images of xenograft tumors from HGC-27 cells (OENC-MMP2, OE-MMP2, OE-MMP2+OE-FBXW7) in nude mice, illustrating size variations (n = 5 per group). **(B)** Tumor weight quantification, showing OE-MMP2+OE-FBXW7 tumors are significantly heavier than OE-MMP2 alone. **(C)** Tumor volume measurement, confirming enhanced growth with FBXW7 overexpression in MMP2-overexpressing cells. **(D)** The protein expression of CoL-1 was detected by immunohistochemistry in the MMP2 overexpression group and the control group. **(E, F)** The protein expression of FBXW7 **(E)** and MMP2 **(F)** was detected through immunohistochemical experiments in the animal experimental tissue sections of solid tumors. The bar chart represents the quantitative analysis values of FBXW7 and MMP2. **(G)** A mechanistic model of FBXW7-MMP2 axis by regulating gastric cancer formation is proposed.

To elucidate the *in vivo* functional significance of the FBXW7-MMP2 axis, we established subcutaneous xenograft tumor models in nude mice using HGC-27 cells stably overexpressing MMP2 alone, empty vector (Control), or both MMP2 and FBXW7.Representative images and quantitative analyses revealed that tumors in the OE-MMP2 group exhibited the largest volume and weight, whereas co-overexpression of FBXW7 significantly attenuated MMP2-induced tumor growth (**Figures 7A–C**). Firstly, the protein expression of collagen I (CoL-1) was detected in MMP2 overexpression mouse tissues by immunohistochemistry in solid tumors derived from animal experiments. The results showed that overexpression of MMP2 did promote collagen degradation. This finding provided direct evidence that MMP2 drives extracellular matrix remodeling and promotes gastric carcinogenesis (**Figure 7D**). Subsequently, immunohistochemical staining confirmed that the two proteins were successfully overexpressed in nude mice. Overexpression of MMP2 protein could promote the formation of gastric cancer, but overexpression of FBXW7 could reverse the process of gastric cancer formation mediated by MMP2 overexpression (**Figures 7E, F**). These findings corroborate our *in vitro* data, demonstrating that FBXW7 acts as a negative regulator to mitigate MMP2-driven gastric cancer progression. In summary, our findings demonstrate that FBXW7 functions as a tumor suppressor in gastric cancer by promoting ubiquitin-mediated degradation of MMP2, thereby limiting its oncogenic potential (**Figure 7G**).

## 4 Discussion

GC metastasis continues to pose a significant clinical challenge, largely due to the heterogeneity of tumors and the intricate molecular mechanisms that drive disease progression. MMP2, a pivotal enzyme involved in the degradation of the ECM, has been associated with unfavorable prognoses in patients with GC (21–24). This study elucidates a novel mechanism in which the ubiquitin ligase FBXW7 inhibits GC progression by facilitating the ubiquitination-dependent degradation of MMP2, thereby providing new insights into the interactions between the stroma and tumor in GC.

ScRNA-seq technology provides a powerful approach for resolving transcriptomic heterogeneity and complexity at single-cell resolution, thereby enabling detailed analysis of the composition and functional states of distinct cell types across tissues, organs, and whole organisms (25–28). ScRNA-seq has been instrumental in elucidating the formation and cellular heterogeneity of GC from various perspectives. For example, Zhang et al. employed scRNA-seq to examine patients with precancerous gastric cancer and early gastric cancer (EGC) lesions, constructing single-cell atlases that offered novel insights into the transformation of precancerous lesions into early-stage GC (29). Similarly, Wang et al. utilized scRNA-seq to conduct a single-cell transcriptomic analysis of peritoneal carcinomatosis in 15 GC patients, creating an atlas that provides a crucial theoretical foundation for understanding the formation, progression, and patient stratification of gastric adenocarcinoma (30).

MMP2 was enriched in the fibroblast cluster annotated as universal fibroblasts (UFs). We use UF as a dataset-specific transcriptional label, consistent with the cross-tissue fibroblast states reported by Buechler et al. (31),. Because CAF nomenclature should be linked to functional evidence (32), we do not regard UFs as an established functional CAF subtype in gastric cancer. The increased abundance of the UF-like cluster and its MMP2 enrichment suggest that this fibroblast state may represent a stromal source of MMP2. However, the present single-cell data do not establish lineage conversion or the cellular transfer route of MMP2 protein.

Our group has long been committed to studying the upstream and downstream mechanism of FBXW7 in gastric cancer progression and peritoneal metastasis. We have previously confirmed that FBXW7 can regulate the progression and peritoneal metastasis of gastric cancer by regulating the expression of Cyclin E, c-MYC, BGN and VDAC3 proteins. We demonstrated that FBXW7 is regulated by lncRNAs (BDNF-AS (14), SEMA3B-AS1 (15)) in an animal model and is involved in the mechanism of ferroptosis of gastric cancer cells and peritoneal metastasis of gastric cancer. Combined with our research group and existing studies, we found that FBXW7 forms a complex and extensive regulatory network in gastrointestinal tumors by regulating various signaling pathways and substrate proteins. Therefore, strategies targeting FBXW7 may provide potential therapeutic approaches for cancer therapy. Although there is no direct targeted FBXW7 drugs, based on the existing research has proposed the promising treatment strategies (33), future treatment might focus on through targeted FBXW7 upstream regulatory factors (such as: LncRAN, micrornas and ERK), or downstream cancer by inhibiting protein (such as BGN, VDAC3, and C - MYC, etc.) to restore or replace FBXW7 expression (34), above all, FBXW7 has high clinical transformation value.

In this study, we provide new insights into the FBXW7-MMP2 regulatory axis in gastric cancer. FBXW7, the substrate-recognition component of the SCF (SKP1-CUL1-F-box) E3 ubiquitin ligase complex, interacts with MMP2 and promotes its polyubiquitination, thereby directing MMP2 toward proteasomal degradation. This mechanism aligns with FBXW7’s recognized role as a tumor suppressor, as evidenced by its regulation of other oncoproteins such as c-Myc and NOTCH. Our functional assays, including the overexpression of FBXW7 in gastric cancer cell lines, demonstrated a significant reduction in MMP2 protein levels, with no notable changes in *MMP2* mRNA levels. This effect was reversed by the application of the proteasome inhibitor MG132, conclusively establishing that FBXW7 modulates MMP2 at the post-translational level.

From a clinical and translational standpoint, our findings demonstrate significant potential. In clinical samples, we identified a notable negative correlation between the expression levels of FBXW7 and MMP2. Elevated MMP2 expression was linked to a markedly reduced 5-year survival rate in patients with gastric cancer, as confirmed by Kaplan-Meier analysis (*P* < 0.05). This correlation is further corroborated by data from extensive databases such as TCGA, as well as our own tissue chip immunohistochemistry results. These findings suggest that the FBXW7-MMP2 axis may serve as an independent prognostic marker for patients with gastric cancer. Clinically, the concurrent assessment of FBXW7 methylation status (given the prevalence of epigenetic silencing of FBXW7 in gastric cancer) alongside MMP2 protein expression levels could enhance the accuracy of postoperative recurrence risk stratification in these patients.

Regarding therapeutic targets, current MMP inhibitors are limited by their lack of cell specificity, which frequently results in significant side effects. In contrast, the FBXW7-mediated degradation of MMP2 represents a highly specific endogenous process. Our *in vivo* experiments utilizing xenograft tumor models demonstrated that overexpression of FBXW7 can significantly inhibit MMP2-driven tumor growth, resulting in an approximate 60% reduction in tumor volume. This finding provides a compelling rationale for the development of FBXW7-based therapeutic strategies. One potential approach involves the development of small-molecule PROTACs (proteolysis-targeting chimeras) to enhance FBXW7 activity. Alternatively, employing epigenetic modifiers to reverse the silencing of FBXW7 in gastric cancer cells represents another promising strategy.

The predicted MMP2-PECAM1 pair may be relevant to vascular remodeling because MMP2 supports endothelial tube formation (35), whereas PECAM1 participates in endothelial junctional signaling and leukocyte transendothelial migration (36, 37). Thus, this predicted interaction could be associated with endothelial behavior and immune-cell trafficking, but direct signaling through this axis remains to be experimentally tested. The effects of the FBXW7-MMP2 axis on macrophage polarization, AKT/mTOR signaling, tumor metabolism, and immunotherapy response were not examined in this study and therefore remain hypotheses for future investigation.

Within the expansive domain of cancer research, numerous avenues warrant further investigation. A pertinent area of inquiry involves elucidating whether FBXW7 interacts with other ubiquitin ligases, such as NEDD4 or CUL3, in the modulation of MMP2. It is plausible that compensatory degradation pathways exist for MMP2, and a deeper understanding of these mechanisms could offer a more holistic perspective on MMP2 regulation in gastric cancer. From an evolutionary biology standpoint, examining the positive selection pressures exerted on the FBXW7-MMP2 axis during gastric cancer metastasis could yield valuable insights into the evolutionary dynamics of tumor progression. Employing methodologies such as tumor evolutionary tree analysis may facilitate the identification of critical mutations or alterations within the FBXW7-MMP2 axis that correlate with more aggressive tumor phenotypes. Empirical data from clinical samples reveal an inverse correlation between FBXW7 expression and MMP2 levels, with elevated MMP2 expression linked to diminished 5-year survival rates. This study underscores the FBXW7-MMP2 axis as a pivotal regulator of tumor microenvironment remodeling in gastric cancer, wherein FBXW7 mitigates invasion and metastasis through the ubiquitin-mediated degradation of MMP2.

These findings not only identify a promising therapeutic target but also reshape our understanding of stromal protease homeostasis, shifting the paradigm from focusing on enzymatic activity modulation to emphasizing protein lifecycle control. Future research should prioritize the development of spatiotemporally precise intervention strategies to address the challenges posed by the pleiotropic substrate specificity of FBXW7. However, the study has certain limitations: the single-cell RNA-seq cohort comprised only three pairs of gastric cancer and normal tissues, which restricts the generalizability of the characterization of cancer-associated fibroblast (CAF) subtypes. Additionally, the lack of spatial transcriptomic data limits our understanding of the anatomical distribution of MMP2+ CAFs and their interactions within the microenvironment. Although subcutaneous xenograft models validated the FBXW7-MMP2 axis, orthotopic and metastatic models are necessary to fully address the complexity of gastric cancer metastasis. Further research is needed to elucidate the upstream regulators of FBXW7 and its specificity for MMP2 in CAFs.

## Data Availability

The datasets presented in this study can be found in online repositories. The names of the repository/repositories and accession numbers can be found in the article/**Supplementary Material**.

## Supplementary material

Supplementary Table 1 Clinicopathologic features of three patients with single cell sequencing.

Supplementary Table 2 Expression of MMP2 in STAD based on Sample types.

Supplementary Table 3 Differential expression of MMP2 in cancer and paracancer.

Supplementary Table 4 FBXW7-interacting proteins identified by co-immunoprecipitation and mass spectrometry.
