# Supplementary material for: FBXW7 suppresses gastric cancer development and progression via ubiquitination-mediated degradation of MMP2
Source: Front Immunol. 2026 Aug 4;17:1877833. doi: 10.3389/fimmu.2026.1877833 (PMC13481373; doi:10.3389/fimmu.2026.1877833)
Supplement: Supplementary file 1 [file DataSheet1.pdf]

Supplementary Table

Supplementary Table 1: The Clinical data of patients with single cell sequencing

Patient ID	Sex	Age (years)	Histopathological differentiation	Site	TNM Stage	Nerve Invasion	Vascular Invasion
1	M	73	Poorly differentiated	Gastric body	T4aN2M0	Yes	Yes
2	M	76	Moderately to poorly differentiated	Gastric body	T4bN0M0	Yes	Yes
3	M	62	Moderately differentiated	Gastric antral	T4N1M0	No	No

Supplementary table-2：Expression of MMP2 in STAD based on Sample types

TCGA samples	Series 1				
	low	q1	median	q3	high
**Normal (n=34)**	3.497	64.386	95.283	116.695	215.285
**Primary tumor (n=415)**	5.594	60.755	116.309	215.186	543.738

Supplementary table-3: Differential expression of MMP2 in cancer and paracancer

—	n	MMP2 expression		Chi-square Value	p value
		High	Low		
Cancer	89	23	66	8.633	0.003
Para-cancer	81	7	74		

Supplementary table-4: The interaction poteins between FBXW7 and MMP2 in HGC-27 cells through co-immunoprecipitation and mass spectrometry analyses

prot_hit_num	prot_acc	prot_score	emPAI
1	sp|P04264|K2C1_HUMAN	127	0.05
2	sp|A0A075B6S6|KVD30_HUMAN	86	0.26
3	sp|P13645|K1C10_HUMAN	68	0.18
4	sp|Q969H0|FBXW7_HUMAN	65	0.04
5	sp|Q9Y3Y2|CHTOP_HUMAN	55	0.13

6	sp|Q4G0S4|C27C1_HUMAN	52	0.05
7	sp|Q15040|JOS1_HUMAN	50	0.14
8	sp|Q9P267|MBD5_HUMAN	48	0.02
9	sp|P02533|K1C14_HUMAN	47	0.06
10	sp|Q7Z3Y9|K1C26_HUMAN	47	0.06
11	sp|Q7Z3Z0|K1C25_HUMAN	47	0.07
12	sp|Q9P2D7|DYH1_HUMAN	40	0.01
13	sp|Q9C0C9|UBE2O_HUMAN	40	0.02
14	sp|P42892|ECE1_HUMAN	38	0.04
15	sp|Q00839|HNRPU_HUMAN	38	0.04
16	sp|Q8WYL5|SSH1_HUMAN	38	0.03
17	sp|Q8IXT2|DMRTD_HUMAN	38	0.08
18	sp|Q8NFR7|CC148_HUMAN	38	0.05
19	sp|Q8NCE0|SEN2_HUMAN	37	0.06
20	sp|P35908|K22E_HUMAN	37	0.1
21	sp|Q6ZR08|DYH12_HUMAN	36	0.01
22	sp|Q96EU6|RRP36_HUMAN	36	0.11
23	sp|Q96S53|TESK2_HUMAN	36	0.05
24	sp|O00755|WNT7A_HUMAN	35	0.08
25	sp|Q5VTD9|GFI1B_HUMAN	35	0.09
26	sp|Q99456|K1C12_HUMAN	34	0.06
27	sp|Q5XKR4|OTP_HUMAN	34	0.1
28	sp|O60242|AGRB3_HUMAN	34	0.02
29	sp|Q9UKT7|FBXL3_HUMAN	34	0.07
30	sp|Q9ULI0|ATD2B_HUMAN	34	0.02
31	sp|Q6PL18|ATAD2_HUMAN	34	0.02
32	sp|Q8TF76|HASP_HUMAN	34	0.04
33	sp|O43865|SAHH2_HUMAN	33	0.05
34	sp|P48668|K2C6C_HUMAN	33	0.05
35	sp|P02538|K2C6A_HUMAN	33	0.05
36	sp|Q01546|K22O_HUMAN	33	0.05
37	sp|Q99570|PI3R4_HUMAN	33	0.02
38	sp|P98077|SHC2_HUMAN	33	0.05
39	sp|P13647|K2C5_HUMAN	32	0.05
40	sp|Q9P2K9|DISP3_HUMAN	32	0.02
41	sp|Q7L3B6|CD37L_HUMAN	32	0.08
42	sp|Q70JA7|CHSS3_HUMAN	32	0.03
43	sp|Q9Y2H8|ZN510_HUMAN	32	0.04
44	sp|Q9NRC6|SPTN5_HUMAN	32	0.01
45	sp|Q02410|APBA1_HUMAN	32	0.04
46	sp|Q8N0X4|CLYBL_HUMAN	32	0.09
47	sp|Q5T7P8|SYT6_HUMAN	31	0.06
48	sp|Q5TAP6|UT14C_HUMAN	31	0.04
49	sp|O43559|FRS3_HUMAN	31	0.06

50	sp|P38432|COIL_HUMAN	31	0.05
51	sp|Q12904|AIMP1_HUMAN	31	0.1
52	sp|Q9NUZ1|ACOXL_HUMAN	31	0.05
53	sp|P25100|ADA1D_HUMAN	31	0.05
54	sp|Q6P597|KLC3_HUMAN	31	0.06
55	sp|Q96BR6|ZN669_HUMAN	31	0.06
56	sp|P0DOX7|IGK_HUMAN	31	0.14
57	sp|Q8IVT5|KSR1_HUMAN	31	0.03
58	sp|Q2M3C7|SPKAP_HUMAN	31	0.02
59	sp|Q9H9D4|ZN408_HUMAN	31	0.04
60	sp|Q9NQT8|KI13B_HUMAN	30	0.02
61	sp|P13798|ACPH_HUMAN	30	0.04
62	sp|Q7LBC6|KDM3B_HUMAN	30	0.02
63	sp|Q05BV3|EMAL5_HUMAN	30	0.01
64	sp|Q05940|VMAT2_HUMAN	30	0.06
65	sp|Q9P2D8|UNC79_HUMAN	29	0.01
66	sp|Q9H3U1|UN45A_HUMAN	29	0.03
67	sp|Q96QE4|LR37B_HUMAN	29	0.03
68	sp|Q15058|KIF14_HUMAN	29	0.02
69	sp|Q15149|PLEC_HUMAN	29	0.01
70	sp|Q68DH5|LMBD2_HUMAN	29	0.04
71	sp|Q9H5Q4|TFB2M_HUMAN	29	0.07
72	sp|P11388|TOP2A_HUMAN	29	0.02
73	sp|Q15417|CNN3_HUMAN	29	0.09
74	sp|Q8WXD0|RXFP2_HUMAN	28	0.04
75	sp|O95206|PCDH8_HUMAN	28	0.03
76	sp|A6NCS4|NKX26_HUMAN	28	0.1
77	sp|O75449|KTNA1_HUMAN	28	0.06
78	sp|O75962|TRIO_HUMAN	27	0.01
79	sp|Q8NDH2|CC168_HUMAN	27	0.01
80	sp|P08253|MMP2_HUMAN	27	0.04
81	sp|B5ME19|EIFCL_HUMAN	27	0.03
82	sp|P43378|PTN9_HUMAN	26	0.05
83	sp|O15211|RGL2_HUMAN	26	0.04
84	sp|Q9Y2K7|KDM2A_HUMAN	26	0.02
85	sp|Q9UNX4|WDR3_HUMAN	26	0.03
86	sp|Q9HB55|CP343_HUMAN	26	0.06
87	sp|P16157|ANK1_HUMAN	26	0.02
88	sp|P02768|ALBU_HUMAN	26	89
89	sp|Q6ZS81|WDFY4_HUMAN	25	0.01
90	sp|Q9UPQ9|TNR6B_HUMAN	25	0.02
91	sp|Q53GS7|GLE1_HUMAN	25	0.04
92	sp|Q7Z5J1|DHI1L_HUMAN	24	0.1
93	sp|A6NNT2|CP096_HUMAN	24	0.03

94	sp|Q96NE9|FRMD6_HUMAN	24	0.05
95	sp|A7E2Y1|MYH7B_HUMAN	24	0.01
96	sp|Q8WZ64|ARAP2_HUMAN	24	0.02
97	sp|Q9H2C8|O51V1_HUMAN	24	0.09
98	sp|Q6ZS17|RIPR1_HUMAN	23	0.02
99	sp|Q5T2S8|ODAD2_HUMAN	23	0.03
100	sp|P0C091|FREM3_HUMAN	23	0.01
101	sp|P20929|NEBU_HUMAN	22	0
102	sp|Q9H720|PG2IP_HUMAN	22	0.04
103	sp|Q99590|SCAFB_HUMAN	22	0.02
104	sp|Q8IX07|FOG1_HUMAN	22	0.03
105	sp|Q6ZNA5|FRRS1_HUMAN	21	0.05
106	sp|P42356|PI4KA_HUMAN	21	0.01
107	sp|Q71F56|MD13L_HUMAN	21	0.01
108	sp|Q9UN79|SOX13_HUMAN	20	0.05
109	sp|Q16384|SSX1_HUMAN	19	0.16
110	sp|Q5VT25|MRCKA_HUMAN	19	0.02
111	sp|Q16739|CEGT_HUMAN	18	0.07
112	sp|P16150|LEUK_HUMAN	17	0.08
113	sp|Q569K4|Z385B_HUMAN	16	0.06
114	sp|Q9H607|OCEL1_HUMAN	16	0.11
115	sp|P40145|ADCY8_HUMAN	16	0.02
116	sp|P14649|MYL6B_HUMAN	14	0.15
117	sp|O60825|F262_HUMAN	14	0.06
118	sp|Q9Y543|HES2_HUMAN	13	0.18
